# Validity, reliability and cut-offs of the Patient Health Questionnaire-9 as a screening tool for depression among patients living with epilepsy in Rwanda

**DOI:** 10.1371/journal.pone.0234095

**Published:** 2020-06-12

**Authors:** Fidèle Sebera, Joao Ricardo Nickenig Vissoci, Josiane Umwiringirwa, Dirk E. Teuwen, Paul E. Boon, Peter Dedeken

**Affiliations:** 1 CARAES Neuropsychiatric Hospital, Ndera, Kigali, Rwanda; 2 Centre Hospitalier Universitaire Kigali (CHUK), Kigali, Rwanda; 3 Department of Surgery, Duke University Medical School, Durham, NC, United States of America; 4 Duke Global Health Institute, Duke University, Durham, NC, United States of America; 5 UCB Pharma, Brussels, Belgium; 6 Ghent University Hospital, Ghent, Belgium; 7 Heilig Hart Hospitaal, Lier, Belgium; Monash University, AUSTRALIA

## Abstract

**Background:**

Patients with epilepsy (PwE) have an increased risk of active and lifetime depression. Two in 10 patients experience depression. Lack of trained psychiatric staff in low- and middle-income countries (LMIC) creates a need for screening tools that enable detection of depression in PwE. We describe the translation, validity and reliability assessment of the Patient Health Questionnaire-9 (PHQ-9) as a screening tool for depression among PwE in Rwanda.

**Method:**

PHQ-9 was translated to Kinyarwanda using translation-back translation and validated by a discussion group. For validation, PwE of ≥15 years of age were administered the PHQ-9 and Hamilton Depression Rating Scale (HDRS) by trained psychiatry staff at Visit 1. A random sample of 20% repeated PHQ-9 and HDRS after 14 days to assess temporal stability and intra-rater reliability. Internal structure, reliability and external validity were assessed using confirmatory factor analysis, reliability coefficients and HDRS-correlation, respectively. Maximal Youden’s index was considered for cut-offs.

**Results:**

Four hundred and thirty-four PwE, mean age 30.5 years (SD ±13.3), were included of whom 33.6%, 37.9%, 13.4%, and 15.1% had no, mild, moderate and severe depression, respectively. PHQ-9 performed well on a one-factor model (unidimensional model), with factor loadings of 0.63–0.86. Reliability coefficients above 0.80 indicated strong internal consistency. Good temporal stability was observed (0.79 [95% CI: 0.68–0.87]). A strong correlation (R = 0.66, p = 0.01) between PHQ-9 and HDRS summed scores demonstrated robust external validity. The optimal cut-off for the PHQ-9 was similar (≥5) for mild and moderate depression and ≥7 for severe depression.

**Conclusion:**

PHQ-9 validation in Kinyarwanda creates the capacity to screen PwE in Rwanda at scores of ≥5 for mild or moderate and ≥7 for severe depression. The availability of validated tools for screening and diagnosis for depression is a forward step for holistic care in a resource-limited environment.

## Introduction

Depression and epilepsy are each associated with a significant burden of disease globally, and are known to occur together. In fact, depression is the most frequent psychiatric comorbidity reported by patients with epilepsy (PwE). Compared with the general population, PwE have a significantly increased risk of active and lifetime depression [1, 2]. Depression has been shown to exacerbate adverse events associated with antiepileptic drugs, such as suicidal ideation; further, patients with depression experience increased levels of stigma [1, 3–7]. Prevalence of active depression in PwE ranged between 14.6% and 23.1% [2, 8, 9]. Up to 1/3 of PwE may experience a major depressive episode in their lifetime [9].

Although depression has a key position in holistic care for PwEs, many healthcare professionals tend to focus attention predominantly on the medical aspects of epilepsy, often neglecting the psychosocial burden of the disease [10]. Psychiatric comorbidities, therefore, remain under-recognised and undertreated, especially in low- and middle-income countries (LMICs) such as Rwanda [5, 11, 12]. Both diseases are subject to a large diagnosis and treatment gap, and are considered to have a burdensome stigma. This is particularly concerning if the burden of both epilepsy and depression are greatly underestimated or if the prevalence of epilepsy is high. For instance, depressive disorders greatly impact disability in sub-Saharan Africa, and are considered a leading cause of years lost due to disability [13]. In Rwanda, the prevalence of epilepsy is up to 49 per 1,000 individuals [14], amongst the highest in Africa. Moreover, the prevalence of depression in PwE is higher in Africa than in developed countries [15–19].

Central to addressing the gap in depression care in PwE in Africa is the ability to screen patients living with or at risk of depression. A number of screening tools are available to enable healthcare providers to identify depression among PwE, including the Patient Health Questionnaire-9 (PHQ-9).

The PHQ-9 is the nine-item depression module of the full PHQ, the patient-administered version of the Primary Care Evaluation of Mental Disorders (PRIME-MD) screening tool, which is administered by healthcare providers [20]. The PHQ-9 follows the diagnostic criteria for depression of the Diagnostic and Statistical Manual of Mental Disorders, Fourth Edition (DSM-IV). It can be used as an algorithm to monitor and screen for major depressive disorder (MDD) [20]. The PHQ-9 has been widely studied in developed countries, with optimal cut-off scores to identify MDD varying from 8 to 11 points [21–24]. However, variations of cut-offs have been reported considering the targeted population and setting.

Although developed and validated for diagnosing depression in primary care, the use of PHQ-9 as a screening tool has also been validated in other clinical settings, among patients with different ethnicities and in different countries [25–34]. Some studies reported the applicability of PHQ-9 in sub-Saharan Africa, including Nigeria [35], Ethiopia [36–38], Kenya [39, 40], Malawi [41], Cameroon [42], Uganda [43] and South Africa [44, 45], but none in Rwanda. Psychometric properties of PHQ-9 have been described as a screening tool for depression, and cut-offs of 5–10 were found to be optimal in general populations [35, 38, 42, 43], while cut-offs of 8–9 were optimal in specific populations [36, 41]. Thus only a few countries in Africa have reported the psychometric properties of PHQ-9 for targeted populations and conditions, and how PHQ-9 performs in PwE has not been ascertained. In fact, a recent review revealed only four studies reporting evidence of validity of PHQ-9 for screening for depression in PwE [46]; none were conducted in Africa.

Rwanda is a landlocked country in East Africa and home to 12.63 million inhabitants, mean age 20y, of which less than 20% live in urban areas. A community-based health insurance model provides cost coverage. Its healthcare system operates health centers, health posts, dispensaries, district hospitals and reference hospitals. Access to neurologists is limited with 0.024 neurologists per 100,000 inhabitants. The CARAES Neuropsychiatric Hospital in Ndera is a reference hospital for mental health, both psychiatry and neurology in- and outpatient care. Of the 21,690 neurology outpatient consultations performed in 2018 at the center, epilepsy accounted for 84.6%.

In the current study, the steps taken for the translation, and subsequent assessment of validity and reliability of PHQ-9 as a tool for screening for depression among PwE in Rwanda, are described.

## Methods

The study was conducted over a 4-month period at the Neuropsychiatric Hospital in Ndera, just outside of Kigali, Rwanda. The study was approved by the Institutional Review Board of the Rwanda National Health Research Committee and the National Ethics Committee. Written informed consent was obtained from study participants before data collection. For patients aged <18y, an additional assent form was signed by the caregiver.

### Study participants

PwE with a clinically confirmed diagnosis by a neurologist and ≥15 years of age, attending the neurology outpatient department of the Neuropsychiatric Hospital, were consecutively enrolled in order of attendance when providing consent. Patients who had a concomitant clinical diagnosis of depression, or reported signs and symptoms of depression, were included. However, those with other psychiatric morbidities were excluded. Presence of co-morbid depression or depressive symptoms as well as other psychiatric diseases was based on assessment by the treating physician following clinical interview.

### Procedure

At the first visit, trained medical nurses and healthcare providers administered the PHQ-9. On the same day, the Hamilton Depression Rating Scale (HDRS), validated in Kinyarwanda for diagnosis of depression, was also administered by trained psychiatry nurses, psychologists and psychiatrists. Investigators administering HDRS were blinded throughout the study to the results of PHQ-9 and *vice versa*. The Kinyarwanda version of the HDRS was used as the comparator/gold standard measure of symptom severity.

A randomly selected sample of 20% of all participants returned after 14 days for a follow-up assessment, and completed the PHQ-9 and HDRS again, administered by the same investigator as on the first visit. This enabled evaluation of its temporal stability and intra-rater reliability. All responses were collected through electronic data capturing and entered into an SQL database.

### Sample size calculation

Sample size was estimated using both Gorsuch’s rule and sample sizes from Comrey and Lee. Gorsuch’s rule requires a sample size of five times the number of questions assessed, a total of 36 in this study, resulting in minimal sample of 180 patients. Comrey and Lee consider a sample size >300 as good and >500 as very good. Based on recruitment feasibility at the center, we aimed for a sample size of 400 patients.

### Instruments

#### Translation and adaptation of PHQ-9

Coordinated by the Integration of HIV Care into Mental Healthcare Services Technical Working Group, the questionnaire was translated from English to Kinyarwanda in 2011. The team was supervised by the International Center for HIV/AIDS Program (ICAP) of the University of Columbia (USA), the Rwanda Biomedical Center/Mental Health division, and the University of Rwanda. A structured approach using a back-translation technique combined with bilingual technique was used [47]. A discussion group, including 10 doctors and psychologists with experience in depression, achieved consensus on the wording of the translated PHQ-9, addressing five major dimensions for cross-cultural equivalence defined by Flaherty [48].

#### HDRS

The HDRS has been used for assessing severity of depression, changes in severity over time, and treatment efficacy, with good overall levels of internal consistency, inter-rater and test–retest reliability [49–51]. The HDRS was used as our gold standard anchor to evaluate the screening ability of the Rwandan adapted PHQ-9. The HDRS was translated at the CARAES Neuropsychiatric Hospital in Ndera, Rwanda, in 2013 and found to be associated with reliable internal consistency (Cronbach’s alpha = 0.96) [18].

In a previous analysis, we determined a single HDRS cut-off point for diagnosis of depression in a Rwandan population. This showed that a score of 17 was the optimal cut-off point to diagnose an MDD, based on expert diagnostic assessment. Since we did not establish cut-off points based on intervals of depression severity, for the current study we compared the PHQ-9 diagnostic ability against international standard intervals suggested in the literature (Table 1) [52], in addition to the single Rwandan cut-off defined in our previous study.

**Table 1:** HDRS Depression screening cut-off points.

HDRS interval	Depression level [52]
0–7	Absence of depression
8–16	Mild
17–23	Moderate
≥24	Severe
HDRS cut-off Rwanda	Moderate and severe depression in Rwandan population
>17	Moderate and severe depression

Abbreviation: HDRS, Hamilton Depression Rating Scale.

### Data analysis

Sociodemographic data are presented as means with standard deviations, medians with the interquartile range, or absolute and relative frequencies. All analyses were conducted with R Language for Statistical Computing v. 3.5 using the lavaan and receiver operating characteristic (ROC) packages (R foundation, Vienna) [53]. Given a small number of patients aged <18, we performed a post-hoc sensitivity analysis excluding those.

#### Evidence of reliability

As a first step in determining the reliability of the translated PHQ-9, its internal consistency was evaluated. This can be assessed by several coefficients, each with its strengths and limitations [54, 55]. For this study, we report the Cronbach’s alpha and Composite Reliability (CR), with values above 0.70 considered adequate.

Temporal stability was first assessed by calculating the intra-class correlation (ICC) between the scores of the PHQ-9 collected at two time points separated by 14 days. ICC values >0.8 are considered good and values >0.9 are exceptional [56].

#### Evidence of validity based on internal structure

To test the internal structure of the PHQ-9, network analysis and confirmatory factor analysis (CFA) was conducted.

For network analysis, graphs of undirected weighted networks were constructed based on a polychoric correlation matrix of PHQ-9 items. Partial correlation coefficients were estimated through nodewise multiple regression, with graphical least absolute shrinkage and selection operator regularisation, or GLASSO [57]. Penalised model selection was made based on extended Bayesian information criteria (EBIC) [58]. Those pairwise partial correlations were depicted as edges connecting nodes. Node size varied according to the mean score for each item. We performed a community structure analysis to identify underlying clusters of items. Community structure analyses are applied to complex networks in which groups of variables are densely interconnected among each other, but sparsely connected to the overall network. The random walks method was incorporated into the Walktrap algorithm, which is suited for weighted networks [59]. With random starts, a limited (in general, three or four) number of steps or “walks” were performed between nodes in such a way that they became “trapped” in high-density subgroups. All analyses were conducted with the R language for Statistical Computing, through the qgraph and igraph packages.

Confirmatory factor analysis was used to determine whether the scores obtained from the nine items of the questionnaire refer to the single construct of depression. Such a single factor, or unidimensional model for PHQ-9, has been reported by others for different populations [37, 60]. The weighted least square means and variance adjusted estimator (WLSMV) was used to test the adequacy of the model. The relationship between each item and depression was determined by its factor loading (values above 0.50 deemed acceptable). Model adjustment, or how well the model fits the data, was evaluated using several goodness-of-fit indices and overall model theoretical interpretation. The fit indices, and the generally accepted reference levels for a good fit (in parentheses), were the following: Chi-square (χ^2^ and p-value), Root Mean Square Error of Approximation (RMSEA, ≤0.05), Tucker–Lewis index (TLI, >0.95), Comparative Fit Index (CFI, ≥0.95) and Average Variance Extracted (AVE, >0.50) [61–63].

#### Evidence of validity based on relationship with other constructs

The validity of the translated PHQ-9 was determined using two methods. For both methods, we used the HDRS as the reference for external validity. First, we evaluated concurrent validity by correlating the scores from PHQ-9 that were associated with the scores of the HDRS. We hypothesised that the PHQ-9 score would positively and strongly predict the HDRS score (R >0.60).

For the second form of external validity, we evaluated the ability of PHQ-9 to identify patients with depressive disorder as defined by the HDRS. We compared the PHQ-9 with two cut-offs defined by the HDRS. Firstly, we evaluated the PHQ-9 parameters to identify a depressive disorder according to the international standards defined as Absence (HDRS <8), Mild (HDRS ≥8), Moderate (HDRS ≥17) and Severe (HDRS ≥24) (Table 1). Secondly, we specified the ability of PHQ-9 to identify moderate to severe depression as defined by the Rwandan cut-off for the HDRS, defined as scores ≥17. We evaluated the screening ability by measuring the sensitivity, specificity and positive and negative predicted values. ROC curves were subsequently generated and the area under the ROC curve (AUROC) was calculated. Using this approach, the optimal cut-off point for the PHQ-9 as a screening tool for the levels of depression as anchored by the HDRS was determined, using both the international cut-offs and the Rwandan cut-off for moderate to severe depression (Table 1). To establish the cut-off, Youden’s index was employed to maximise sensitivity and specificity in detecting patients with moderate to severe depression. As age and level of education may influence PHQ-9 cut-offs, we evaluated the ROC and cut-offs for age groups of <30 years and ≥30 years of age, and by level of education (no schooling/primary schooling and secondary/higher education).

## Results

### Study participants

Four hundred and thirty-four patients participated in the study. Only 2.1% were younger than 18 years of age (Table 2). Up to two-thirds of patients were unmarried. Most participants were employed (59.2%), and more than half had completed secondary education (50.7%). The average age at epilepsy onset was approximately 21 years. Based on the HDRS, 66.4% of patients were diagnosed as having depression.

**Table 2:** Sociodemographic and clinical characteristics of participants.

	Patients (N = 434)
Age (years), mean ± SD	30.5 ± 13.3
Male, n (%)	259 (59.7)
Employed, n (%)	257 (59.2)
Education level, n (%)	
No schooling	29 (6.7)
Primary	185 (42.6)
Secondary	171 (39.4)
Higher education	49 (11.3)
Marital status, n (%)	
Single	283 (69.1)
Married	116 (28.3)
Separated/divorced/widowed	24 (5.8)
Other	2 (0.5)
Not reported (<18 years of age)	9 (2.1)
Age at epilepsy onset (years), mean ± SD	20.8 (13.3)
Seizure type, n (%)*	
Focal	189 (43.5)
Generalised	348 (80.2)
Unknown	11 (2.5)
Depression severity (HDRS), n (%)	
Absence	160 (33.6)
Mild	123 (37.9)
Moderate	71 (13.4)
Severe	63 (15.1)
Incomplete	17 (3.9)
PHQ-9, Mean (SD)	5.8 (5.4)

Abbreviations: HDRS, Hamilton Depression Rating Scale; PHQ-9, Patient Health Questionnaire-9; SD, standard deviation.

*More than one type of seizure can be present in one patient; because of diagnostic limitations, focal to general seizures were considered as possibly generalised.

### Rwandan PHQ-9 characterisation

**Fig 1:**
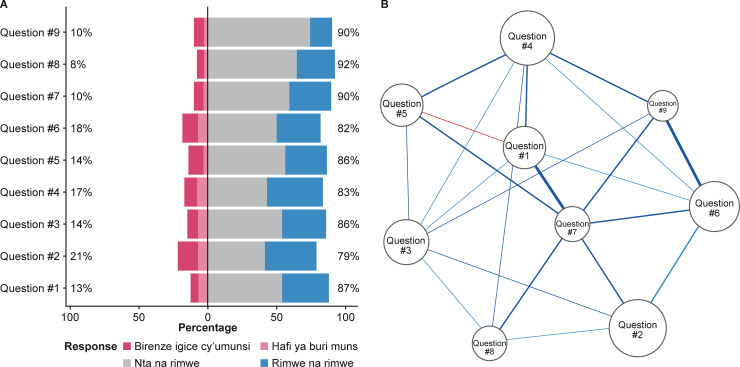
Likert responses distribution (A) and correlation pattern between items (B) of the Rwandan version of the PHQ-9. The size of the nodes (circles) represents the average response in the Likert scale for each item. The thickness of the lines (edges) represents the partial correlation between items. Nta na rimwe = not at all, never; Rimwe na rimwe = several days, sometimes; Birenze igice cy’umunsi = more than half the days; Hafi ya buri munsi = nearly every day.

Overall PHQ-9 score was 5.8 (SD 5.4). All items had a varied distribution of Likert options endorsed (Fig 1A), with higher frequencies for options 3 and 4. Items 2, 4 and 6 had the highest averages among all PHQ-9 items (Fig 1B). Associations between items showed that all items correlated with each other (all correlations had R >0.50). When adjusting for all the correlations (partial correlations) in a network model, items 1 and 5 showed a negative correlation. All items clustered within one community, as expected. Items 1, 3 and 7 had the highest measure of betweenness and expected influence, suggesting that these items are relevant depression symptoms in the behaviour of other depressive symptoms measured using the PHQ-9.

### Internal structure and reliability

**Fig 2:**
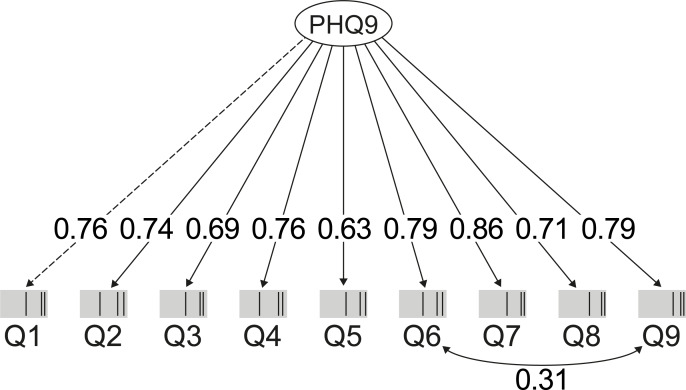
Confirmatory factor analysis models for PHQ-9.

Parallel analysis supported the unidimensional structure of the Rwandan version of the PHQ-9. The one-factor model (unidimensional model) performed well using PHQ-9, and displayed a good fit for the data (Table 3). All nine PHQ-9 items were loaded onto one single factor, with factor loadings for all items in the range 0.63–0.86 (Fig 2). For the reliability of the PHQ-9 items, values above 0.80 were obtained for both reliability coefficients, indicating strong internal consistency (Table 3). Analysis of stability over time in the PHQ-9 assessment through the ICC showed good temporal stability with a score of 0.79 (95% CI: 0.68–0.87) (Table 3).

**Table 3:** Reliability measures, model parameters and goodness-of-fit indicators of the CFA model.

	PHQ-9
*CFA fit indicators*	
X^2^ (Degrees of Freedom)	61.89 (36)
RMSEA (95% CI)	0.06 (0.04–0.08)
Tucker–Lewis Index	0.99
Comparative Fit Index	0.99
*Reliability indicators*	
Cronbach’s alpha (95% CI)	0.87 (0.85–0.89)
Composite reliability	0.97
Temporal stability (95% CI)	0.79 (0.68–0.87)
*Model parameters*	
Factor loadings range	0.63–0.86
Average variance extracted	0.63

Abbreviations: CFA, confirmatory factor analysis; CI, confidence interval

RMSEA, Root Mean Square Error of Approximation.

### Correlation and diagnostics indicators with HDRS

A strong, positive correlation (R = 0.66, p = 0.01) was observed between PHQ-9 and HDRS summed scores, indicating robust external validity and high agreement between both tools. In terms of the diagnostic validity of PHQ-9 relative to HDRS, the PHQ-9 showed good accuracy (area under the curve [AUC] >0.80) in discriminating between participants who did not have depression and those with mild or moderate and severe depression (Table 4).

**Table 4:** Receiver operating characteristic curves of the PHQ-9 scores in relation to HDRS categories: Mild, moderate, severe.

Depression severity	Optimal cut-off	Sensitivity	Specificity	PPV	NPV	AUC
Mild (HDRS >7)	>4	72.4	69.6	79.1	61.2	0.80 (0.74;0.86)
Moderate (HDRS >16)	>4	88.8	59.1	50.6	91.8	0.84 (0.80;0.90)
Severe (HDRS >23)	>6	93.7	65.3	32.4	98.3	0.87 (0.83;0.93)
Rwandan version (HDRS > 17)	>6	84.1	75.1	58.2	92.0	0.86 (0.82;0.90)

Interestingly, the optimal cut-off point for the PHQ-9 was the same for the HDRS groups of mild depression and moderate depression (5 or more). The cut-off for identifying severe depression, as measured by the HDRS was 7 or more (Table 4). The cut-off for the Rwandan version of the HDRS moderate-severe classification was also 7 or more. Detailed values are provided in S1 Table (see Supporting Information). A post-hoc sensitivity analysis, excluding PwE <18y (2.1%), did not affect cut-offs nor validity.

### Subgroup analysis by age and education

Subgroup analyses by age and schooling, factors possibly influencing PHQ-9 cut-offs, showed only small differences between groups in optimal cut-offs (Table 5), demonstrating little influence of these factors.

**Table 5:** Subgroup analysis of optimal cut-offs by age and level of education.

Depression severity	Optimal cut-off	Sensitivity	Specificity	PPV	NPV	AUC
*Less than 30 years of age *						
Mild (HDRS >7)	>4	64.9	71.4	77.8	56.9	0.78 (0.70;0.85)
Moderate (HDRS >16)	>6	70.8	77.7	57.6	86.1	0.79 (0.71;0.87)
Severe (HDRS >23)	>6	91.7	72.7	37.3	98.0	0.89 (0.83;0.95)
*Above 30 years of age *						
Mild (HDRS >7)	>4	75.4	0.68	79.3	0.63	0.80 (0.73;0.86)
Moderate (HDRS >16)	>4	94.2	60.9	56.9	95.1	0.88 (0.82;0.92)
Severe (HDRS >23)	>7	91.2	73.8	41.9	97.6	0.88 (0.82;0.93)
*No education/ Primary education level*						
Mild (HDRS >7)	>5	64.4	86.5	89.7	57.1	0.81
Moderate (HDRS >16)	>6	78.8	74.8	59.1	88.4	0.83 (0.77;0.89)
Severe (HDRS >23)	>7	90.6	74.6	39.2	97.8	0.86 (0.80;0.92)
*Secondary or higher education level*						
Mild (HDRS >7)	>4	74.6	69.0	77.1	65.9	0.80 (0.74;0.86)
Moderate (HDRS >16)	>5	88.2	68.1	57.1	92.3	0.85 (0.80;0.90)
Severe (HDRS >23)	>8	80.6	79.3	40.3	95.9	0.88 (0.83;0.93)

Abbreviations: AUC, area under the curve; HDRS, Hamilton Depression Rating Scale; NPV, negative predictive value; PPV, positive predictive value.

## Discussion

The study was conducted to assess the reliability and validity of the PHQ-9 as a screening tool for depression in a population of Rwandan patients with epilepsy. This study is unique because it addresses the gap of evidence with respect to the psychometric properties of the PHQ-9 and confirms its clinical usefulness in Rwandan PwE. The ROC analysis showed that PHQ-9 thresholds of 5 or more and 7 or more offered the optimal discriminatory power in detecting MDD severity levels (mild, moderate or severe) with acceptable sensitivity, specificity and area under the ROC curve. Overall, results indicated that the tool in Kinyarwanda displayed good psychometric properties in this specific population.

The internal structure of the translated PHQ-9 was consistent with a single-factor model, or unidimensional model, similar to that reported in other sub-Saharan countries and LMICs. The observation that all nine items of the questionnaire load onto a single factor is an indication that the PHQ-9 is measuring a coherent, unitary concept of MDD based on the DSM-IV criteria [33]. This single-factor model has also been reported in other studies conducted in different populations [37, 39, 40, 42, 44], supporting the cultural consistency of this measurement. However, this is the first time that this evaluation has been conducted in sub-Saharan Africa and specifically on a population with epilepsy [33, 37, 60]. All goodness-of-fit indices displayed values for a good fit and did not indicate issues with model identification and fitness.

The translated PHQ-9 was reliable, showing a high degree of internal consistency and temporal stability. Values for all reliability coefficients were >0.80. While results for both tests demonstrated good temporal stability, in the sample that was retested after two weeks, there was more variability in the scores obtained at the two time points when the PHQ-9 was administered. The results were similar to other studies conducted in sub-Saharan Africa [35, 38, 42, 43]. In regard to validity, patients’ scores on the PHQ-9 correlated strongly with their scores from the HDRS. The positive correlation confirmed the instrument’s ability to discriminate levels of MDD in comparison with HDRS scores. Similar results were consistently reported in the literature with diverse populations [64] but this is the first study reporting this validity in patients with epilepsy in sub-Saharan Africa.

Our proposed cut-offs are lower than the score of 10 typically suggested as the cut-off point for depression in a general population in developed countries [65]. However, studies in LMICs in different settings and populations have reported cut-offs for any or mild depression of 5 and severe depression of 10 in Nigerian students and in a Malaysian primary care setting [34, 35, 38, 42, 43].

Cut-offs for the PHQ-9 thus vary across populations [22], differing in clinical setting, concomitant disease and culture, and require population specific values rather than inflexibly adhering to a single cut-off point [20]. Studies with targeted specific patient populations recommend highly variable cut-offs, ranging from 6–8 in chronically ill older patients in the Netherlands [66] to 15 in white female psychiatric in-patients [67]. Other studies have reported values in the range 8–12 in targeted populations including traumatic brain injury [68], HIV [39, 45], cancer [40] and diabetes [69].

Our cut-off of ≥5 for moderate and ≥7 for severe depression in PwE is amongst the lowest of targeted populations and lower compared to cut-offs for PwE of 9 [8] in Canada and 10–15 in the United States [9]. To explain heterogeneity between cut-offs, a meta-analysis was conducted to explore possible factors, which found that blind application of a diagnostic gold standard was a predictive source of heterogeneity [22]. In our study, the administrator of the diagnostic gold standard was blinded to the PHQ-9 results. We also explored possible subgroup differences in our own sample, as education level and age have been suggested to influence PHQ-9 [70]. In our sub-group analysis, no clinically relevant differences for PHQ-9 cut-offs between age groups and educational levels were observed.

Underreporting of depressive symptoms is another probable contributor to lower cut-offs. Firstly, the presence of disease specific symptoms that are difficult to disentangle from somatic symptoms of depression, may lead to underreporting of depressive symptoms by patients. Indeed, improved clinical utility of the Beck Depression Inventory has been observed in HIV patients when somatic items were removed from the questionnaire [70]. Concerning our data, epilepsy is often associated with disease related symptoms or treatment side effects such as fatigue and somnolence, poor concentration or appetite and weight changes, which are all the subject of questions on the PHQ-9. The impact of epilepsy specific symptoms on the performance of depression scales has to our knowledge not been studied and requires more research. Second, differences between populations and cultures need to be considered, such as enacted and self-stigma, which negatively affect help- and care-seeking patterns [71]. Underreporting of depressive symptoms due to social and self-stigma has been documented in war veterans, who reported depressive symptoms up to 4 times more frequently during an anonymous paper questionnaire compared to normal routine healthcare visits [72]. A study from India confirmed a relationship between self-stigma and PHQ-9 scores among college students [73]. In Turkish patients, a stigma score combined for enacted and self-stigma, accounted for 26.2% of the variance in the BDI score [74]. In Rwanda, epilepsy-related enacted stigma is significant as nearly two-thirds of the general population would exclude PwE from school, work and social gatherings [14]. Anticipation of a diagnosis of a mental health disorder such as depression, may thus impact PHQ-9 scoring by PwE experiencing any form of stigma. Clearly, the effects of social and self-stigma on PHQ-9 scoring in PwE has not yet clearly been elucidated. We recommend future research addresses factors that influence the variability of the raw scores and cut-offs, including disease related somatic symptoms and stigma as they probably account for the variability observed in recommended cut-offs.

One limitation of this study is that only patients with epilepsy ≥15 years of age were included. Therefore, cut-offs may not be applicable to paediatric PwE, or to the general Rwandan population. We included too few patients of <18 years of age to perform subgroup analysis on this specific subgroup. Further studies should replicate the scales in other samples and other cultures to confirm the psychometric properties in adolescents.

Another limitation is the comparator chosen for confirmation of depression [46]. The gold standard for diagnosis of depression is the structured interview. In this study, we opted for the HDRS as a diagnostic reference because it has already been validated in Rwanda with established cut-offs for depression diagnosis. Although HDRS is not the most common method used as an anchor for accuracy testing, HDRS specific cut-offs and ranges have been previously validated in relation to a clinical interview and have been extensively reported as an accurate measure for depression [28, 52, 75]. We selected HDRS as an anchor because its use can be scaled in Rwanda to medical professionals working in resource-limited environments. When there is limited availability of trained neurological or psychiatric medical staff to conduct a structured interview for diagnosis of depression, psychometric tools are used to support patient care.

For PwE in Rwanda, PHQ-9 and HDRS tools provide screening and diagnostic capabilities, respectively, and will play a role in closing the diagnosis and treatment gap. The decision of whether to screen for moderate/severe or severe depression only using PHQ-9 cut-offs of ≥5 and ≥7, respectively, will require careful consideration in each healthcare setting (healthpost, healthcare center, district hospital) as screening for and diagnosis of depression will increase the burden on mental healthcare.

## Conclusion

Considering that depressive disorder is a major cause of comorbidity in epilepsy, this cross-cultural validation further develops the capacity to screen for, measure, and treat depressive disorders in patients with epilepsy in Rwanda. Given that a limited number of studies have validated the use of the PHQ-9 in sub-Saharan Africa, our results allow more evidence-based clinical practices. Cut-off scores of 5 and 7 or more for moderate and severe depression were established. In essence, the availability of validated tools for screening and diagnosis for depression in patients with epilepsy in Rwanda is an important step forward in their holistic care in a resource-limited ecosystem.

## Supporting information

S1 Table PHQ-9 screening parameters for mild, moderate and severe depressive disorder as defined by the international HDRS scores. Bolded numbers represent the maximal Youden index. Abbreviations: HDRS, Hamilton Depression Rating Scale; PHQ-9, Patient Health Questionnaire-9; NPV, negative predictive value; PPV, positive predictive value; Sens, Sensitivity; Spec, Specificity.
(DOCX)
