# Supplementary material for: Validity, reliability and cut-offs of the Patient Health Questionnaire-9 as a screening tool for depression among patients living with epilepsy in Rwanda
Source: PLoS One. 2020 Jun 12;15(6):e0234095. doi: 10.1371/journal.pone.0234095 (PMC7292570; doi:10.1371/journal.pone.0234095)
Supplement: S1 Table — Bolded numbers represent the maximal Youden index. Abbreviations: HDRS, Hamilton Depression Rating Scale; PHQ-9, Patient Health Questionnaire-9; NPV, negative predictive value; PPV, positive predictive value; Sens, Sensitivity; Spec, Specificity. (DOCX) [file pone.0234095.s001.docx]

**Supporting Information**

**Table S1. PHQ-9 screening parameters for mild, moderate and severe depressive disorder as defined by the international HDRS scores.**

|  | **Mild depression**  **(HDRS 8-16)** | | | | **Moderate depression (HDRS 17-23)** | | | | **Severe depression (HDRS ≥24)** | | | |
| --- | --- | --- | --- | --- | --- | --- | --- | --- | --- | --- | --- | --- |
|  | **Sens** | **Spec** | **PPV** | **NPV** | **Sens** | **Spec** | **PPV** | **NPV** | **Sens** | **Spec** | **PPV** | **NPV** |
| 0 | 1.00 | 0.00 | 0.58 | - | 1.00 | 0.00 | 0.33 | NA | 1.00 | 0.00 | 0.15 | NA |
| 1 | 0.93 | 0.33 | 0.66 | 0.78 | 0.99 | 0.26 | 0.39 | 0.97 | 1.00 | 0.20 | 0.18 | 1.00 |
| 2 | 0.90 | 0.47 | 0.71 | 0.77 | 0.96 | 0.35 | 0.42 | 0.94 | 1.00 | 0.30 | 0.20 | 1.00 |
| 3 | 0.82 | 0.62 | 0.75 | 0.71 | 0.94 | 0.51 | 0.48 | 0.95 | 1.00 | 0.40 | 0.23 | 1.00 |
| **4** | **0.75** | **0.69** | **0.77** | **0.66** | **0.93** | **0.61** | **0.53** | **0.95** | 0.97 | 0.51 | 0.26 | 0.99 |
| 5 | 0.70 | 0.77 | 0.81 | 0.64 | 0.88 | 0.68 | 0.57 | 0.92 | 0.95 | 0.60 | 0.30 | 0.99 |
| 6 | 0.64 | 0.82 | 0.83 | 0.62 | 0.81 | 0.72 | 0.59 | 0.89 | **0.94** | **0.65** | **0.32** | **0.98** |
| 7 | 0.52 | 0.86 | 0.84 | 0.56 | 0.69 | 0.79 | 0.62 | 0.84 | 0.87 | 0.73 | 0.37 | 0.97 |
| 8 | 0.45 | 0.92 | 0.89 | 0.54 | 0.65 | 0.87 | 0.71 | 0.84 | 0.81 | 0.79 | 0.40 | 0.96 |
| 9 | 0.37 | 0.93 | 0.88 | 0.51 | 0.54 | 0.90 | 0.73 | 0.80 | 0.73 | 0.82 | 0.42 | 0.94 |
| 10 | 0.32 | 0.97 | 0.93 | 0.50 | 0.49 | 0.94 | 0.79 | 0.79 | 0.67 | 0.86 | 0.45 | 0.94 |
| 11 | 0.29 | 0.97 | 0.92 | 0.49 | 0.46 | 0.95 | 0.82 | 0.78 | 0.62 | 0.88 | 0.48 | 0.93 |
| 12 | 0.25 | 0.97 | 0.91 | 0.48 | 0.40 | 0.96 | 0.82 | 0.77 | 0.56 | 0.90 | 0.50 | 0.92 |
| 13 | 0.21 | 0.98 | 0.93 | 0.47 | 0.34 | 0.96 | 0.82 | 0.75 | 0.44 | 0.92 | 0.50 | 0.90 |
| 14 | 0.13 | 0.98 | 0.89 | 0.45 | 0.22 | 0.98 | 0.83 | 0.72 | 0.37 | 0.95 | 0.55 | 0.89 |
| 15 | 0.11 | 0.99 | 0.93 | 0.44 | 0.18 | 0.99 | 0.86 | 0.71 | 0.33 | 0.96 | 0.60 | 0.89 |

Bolded numbers represent the maximal Youden index

Abbreviations: HDRS, Hamilton Depression Rating Scale; PHQ-9, Patient Health Questionnaire-9; NPV, negative predictive value; PPV, positive predictive value; Sens, Sensitivity; Spec, Specificity.
